# Deep sequencing of the T cell receptor β repertoire reveals signature patterns and clonal drift in atherosclerotic plaques and patients

**DOI:** 10.18632/oncotarget.19892

**Published:** 2017-08-03

**Authors:** Zongwei Lin, Shao Qian, Yan Gong, Jianwei Ren, Lixia Zhao, Dongxiao Wang, Xiaowei Wang, Yun Zhang, Zhe Wang, Qunye Zhang

**Affiliations:** ^1^ The Key Laboratory of Cardiovascular Remodeling and Function Research, Chinese Ministry of Education and Chinese Ministry of Health, and The State and Shandong Province Joint Key Laboratory of Translational Cardiovascular Medicine, Qilu Hospital of Shandong University, Jinan, China; ^2^ Division of Endocrinology and Metabolism, Shandong Provincial Hospital Affiliated to Shandong University, Jinan, China; ^3^ Department of Women Health Care, Jinan Maternity and Child Care Hospital, Jinan, China; ^4^ Pharmacy Department of Shandong Medical College, Jinan, China; ^5^ Department of Pharmacy, Chinese PLA General Hospital, Beijing, China; ^6^ Health Division of Guard Bureau, General Staff Department of Chinese PLA, Beijing, China

**Keywords:** T cell receptor β, atherosclerosis, immune repertoire, next-generation DNA sequencing, complement determining region 3, Immunology and Microbiology Section, Immune response, Immunity

## Abstract

The T cell receptor (TCR) β repertoire directly reflects the status of T cell function. Meanwhile, the immune/inflammatory responses regulated by T cells are the critical determinants of atherosclerosis development. However, due to technical limitations, the composition and molecular characteristics of the TCR repertoire in atherosclerotic patients have not been fully elucidated. In the present study, we use powerful immune repertoire sequencing technology to study this issue. Results show that the utilization of V and/or J genes and the diversity of TCRβ repertoire in atherosclerotic plaques are significantly reduced compared to those in the peripheral blood of normal subjects and atherosclerotic patients. The frequencies of the common T cell clones with certain lengths of the complement determining region 3 regions are notably different among all groups. The high-frequency common clones are also increased in the atherosclerotic plaques compared to that in the other two groups. The expansion of several T cell clonotypes (V29-1J2-1, V20-1J1-6, V6-3J2-7 and V11-2J2-2) is validated in atherosclerotic patients. In short, this study reveals that the diversity of TCR β repertoire significantly decreases in atherosclerotic plaques, probably because of the reduced utilization of VJ genes and marked expansion of some T cell subclones. It provides the basis for understanding the roles of T lymphocytes in the pathogenesis of atherosclerosis.

## INTRODUCTION

Atherosclerosis (AS) plays crucial roles in the development of serious cerebro- and cardiovascular diseases. Immuno-inflammatory abnormalities are pivotal factors in the pathogenesis of atherosclerosis, but the mechanism that triggers these changes has not yet been fully elucidated. T cells are the key regulators of immunity and are among the first cells to be recruited in atheroma, participating in all stages of atherosclerosis development. [1-4]. However, T cells are a heterogeneous population and include many subsets which can either promote or suppress inflammation, posing significant challenges to declare the role and mechanism of T lymphocytes in the development of atherosclerosis. Some studies show that atherosclerosis is reduced in immunodeficient mice, and transferring CD4+T cells can reverse the atheroprotective effect of T cell defects [5, 6], but studies on the CD4-deficient ApoE-/- mice observe the opposite effect [7, 8]. Regardless of these complexity, a key point is that during the development of atherosclerosis, the activated/expanded T cells play functions, such as secreting inflammatory factors (IL-6, IL-1β and TNF-α), recruiting inflammatory cells and regulating inflammatory responses [9]. Therefore, revealing the composition and expansion of T cell subtypes in atherosclerotic patients more precisely is the basis for elucidating how the inflammatory response is evoked in atherosclerosis development.

T-cell immune repertoire includes all subtypes of T cell clones within a certain scope. Its diversity and composition directly reflect the status of the immune response and are markedly altered in many diseases, such as ACPA+ synovitis and cancers [10, 11]. However, the reports of the immune repertoires in patients with AS are relatively few. Even in the reported studies, the profiles of T-cell subtypes are also not revealed with sufficient accuracy and resolution. The main reason is that the spectroscopic technology used in the previous studies does not meet the requirements for revealing the actual diversity of the T-cell repertoire [12].

The potential diversity of the T cell receptor (TCR) is extremely astonishing ( > 10^18^), which is far beyond the resolution and sensitivity of many techniques for studying it, such as complement determining region 3 (CDR3) spectratyping. Those techniques can only approximate the diversity of the TCR by some high-frequency clones. In recent years, the development of next-generation DNA sequencing (NGS) technology has provided the ultra-high sensitivity and single-base resolution, which solves, to a large extent, the abovementioned problems. Thus, immune repertoire sequencing (IR-Seq) based on NGS has been widely used in the study of immune repertoire [13]. IR-Seq can simultaneously identify millions of T or B cell clonotypes at single-base resolution, which makes it possible to accurately evaluate the diversity and composition of the immune system.

In the present study, using the powerful IR-Seq technology, we comprehensively analyzed the diversity, composition and molecular characteristics of the TCR β repertoire in the peripheral blood and plaques of patients with AS, including clonotype frequency, CDR3 length distribution and V/J gene utilization. The results revealed the features and drift of T cell clones in patients with AS. Several T cell clonotypes that were significantly expanded in patients with AS were further validated. The present study contributed to the understanding of the roles of T cells in atherosclerosis development and the mechanism of the abnormal immune/inflammatory response in atherosclerosis.

## RESULTS

### The characteristic types and frequency distributions of T cell clones in patients with AS

**Figure 1:**
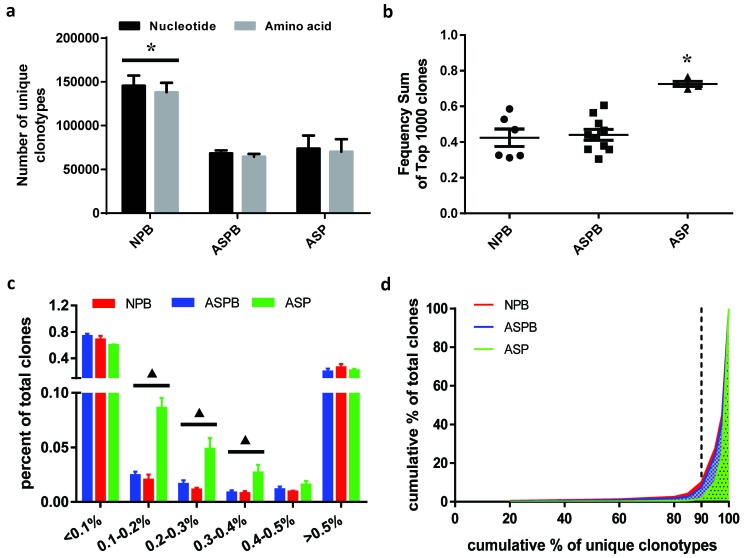
The characteristic types and frequency distributions of T cell clones in patients with atherosclerosis (AS) **a.** At the amino acid and nucleotide levels, the unique clonotypes of the T cells in the peripheral blood of healthy subjects (NPB) were significantly more than those in the peripheral blood (ASPB) and plaques (ASP) of patients with AS. *: *p* < 0.05 *vs*. all other groups. **b.** The sum of the frequency of the top 1000 T cell clones from AS plaques (ASP) was significantly higher than that from the groups of NPB and ASPB. *: *p* < 0.05 *vs*. all other groups. **c.** The percent of total T cell clones in different intervals of clonal frequency was analyzed as described in Methods. The T cell clones in the AS plaques (ASP) were significantly elevated compared to those in the other two groups in certain frequency intervals (0.1-0.4%). ▲: *p* < 0.05 *vs*. NPB and ASPB at the corresponding frequency intervals. **d.** The cumulative percentage of the unique T cell clones was analyzed. The unique T cell clonotypes with high-frequency in the AS plaques (ASP) were more than those in the other two groups (ASPB and NPB).

The T cell repertoires of the atherosclerotic plaques and peripheral blood from healthy subjects and patients with AS, as well as the corresponding sample pools, were analyzed using IR-Seq technology. The demographic information was shown in Additional File Supplementary Table S1. The electrophoresis results of the multiple-PCR products of TCRβ CDR3 region were shown in Additional File Supplementary Figure S1. After filtration, the clean IR-Seq data from each sample exceeded 2GB, and the sequencing depth was approximately 2×10^7^. The rate of the alignment with IMGT database was more than 80% (Supplementary Table S2). There was no significant difference in the ratio of the T cell clones that contained productive rearranged TCRβ genes (namely amino acid clonotypes) to the total T cell clones (nucleotide clonotypes) among all groups. However, the unique clonotypes of the T cells at the amino acid and nucleotide levels were significantly higher in the peripheral blood of healthy subjects than in the other two groups (*p* < 0.05) (Figure 1a). The sum of the frequency of the top 1000 T cell clones in AS plaques was significantly higher than that in the peripheral blood of both normal subjects and patients with AS (*p* < 0.05) (Figure 1b). These results indicated that the number of T cell clonotypes in AS plaques was reduced, but the numbers of T cell clones with high-frequency and in certain frequency intervals (0.1-0.4%) in AS plaques were significantly higher than that in the other two groups (*p* < 0.05) (Figure 1c). The difference of the cumulative percentage of unique T cell clones was not significant between the sample pools of normal subjects and patients with AS. However, more unique T cell clones were high-frequency in the AS plaques (Figure 1d).

### Distribution of CDR3 lengths of common/total clones and the frequency of common clones between individuals

**Figure 2:**
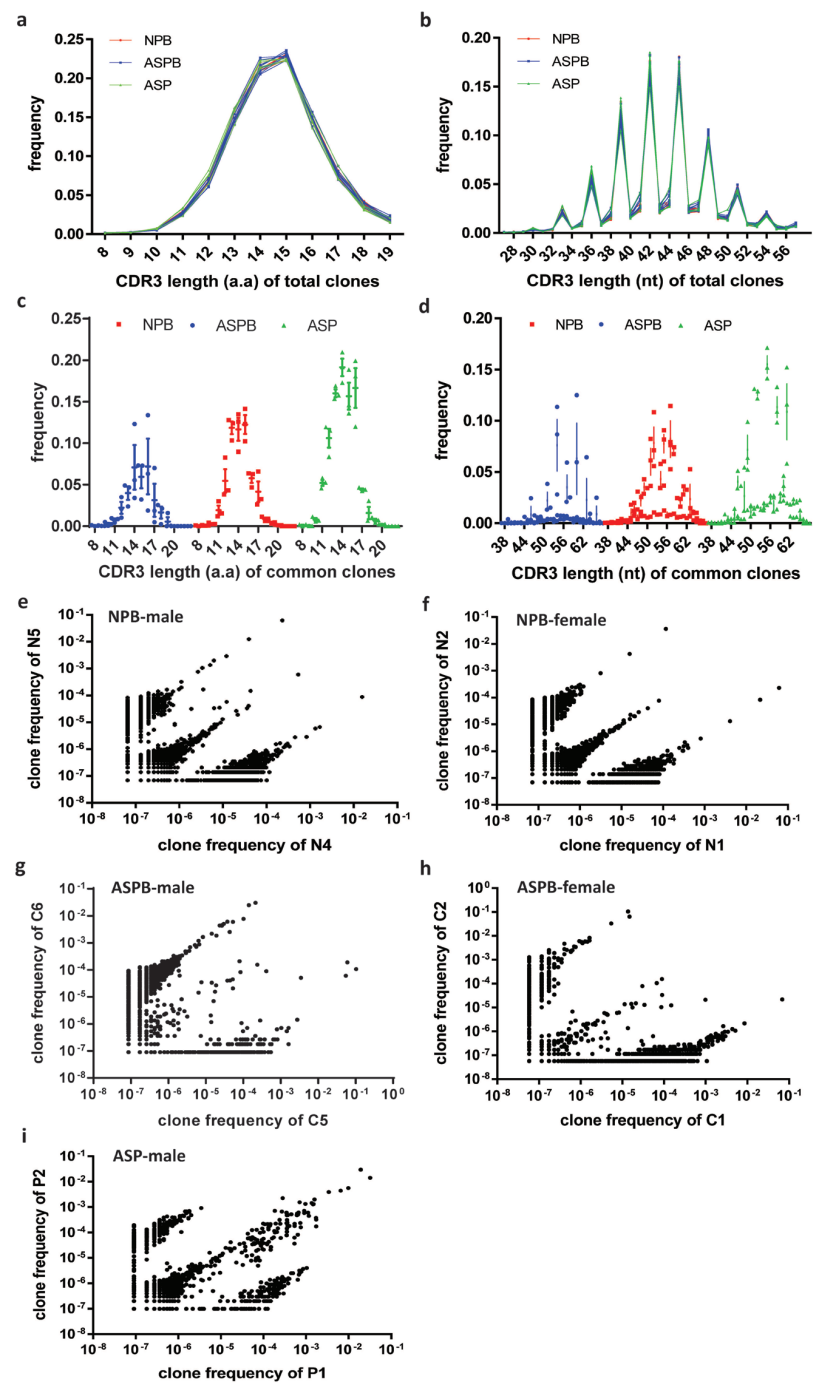
Distribution of CDR3 lengths of common/total T-cell clones and the frequency of common clones between individuals **a.** and **b.** The distributions of CDR3 lengths of the total T-cell clones were not significantly different among the three groups at the amino acid **a.** and nucleotide **b.** levels. All groups showed a normal distribution. **c.** and **d.** The distributions of CDR3 lengths of the common T-cell clones in each group at amino acid **c.** and nucleotide **d.** levels. The frequencies of the common T-cell clones with 13-15 amino acids length were notably higher in AS plaques (ASP) than in the other two groups (NPB and ASPB). The frequencies of the common T-cell clones with certain nucleotides lengths (such as 52, 55 and 58) in ASP were markedly higher than those in the other two groups. **e.**-**i.** The frequency distributions of the common T-cell clones among the different individuals in the same group. The frequency distribution and the number of common T-cell clones in the peripheral blood of normal males **e.** and females **f.** were similar and notably higher than those in the peripheral blood of male **g.** and female **h.** patients with AS. The number of common T cell clones in ASP **i.** were markedly higher than those in the other two groups.

The amino acid and nucleotide length of CDR3 is an important feature of T cell clonal distribution. Our results showed that this feature was not significantly different among each sample of each group (Figure 2a and 2b). At the amino acid and nucleotide levels, the CDR3 lengths of the common clones among different samples in all three groups showed a normal distribution. The frequencies of the common T cell clones with 13-15 amino acids were significantly higher in the AS plaques than in the peripheral blood of the normal subjects and patients with AS (*p* < 0.05) (Figure 2c). Moreover, the frequencies of common T cell clones with certain nucleotide lengths (such as 52, 55 and 58) in the AS plaques were also significantly higher than those in the other groups (*p* < 0.05) (Figure 2d). Next, we further analyzed the common T cell clones among the different individuals in the same group. The results showed that in the peripheral blood of normal males and females, the frequency distributions of common T cell clones were similar and the number of common clones was significantly higher than that in the peripheral blood of the corresponding gender of the patients with AS (Figure 2e-2h). Furthermore, the common T cell clones in the plaques of male patients with AS were markedly elevated compared with that in the peripheral blood of normal male subjects and male patients with AS (Figure 2i).

### Analysis of open reading frame, insertion and amino acid utilization of the T cell clones

**Figure 3:**
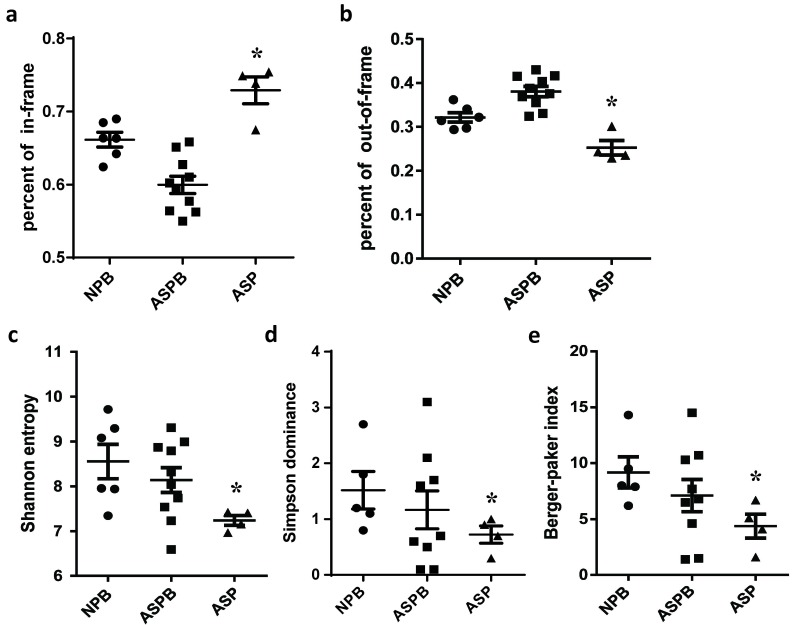
Analysis of the open reading frame and diversity of T cell clones **a.** The proportion of in-frame T-cell clones in the AS plaques (ASP) was significantly higher than that in the other two groups (NPB and ASPB). *: *p* < 0.05 *vs*. all other groups. **b.** The proportion of the out-of-frame T-cell clones in the AS plaques was significantly lower than that in NPB group and ASPB group. *: *p* < 0.05 *vs*. all other groups. **c.**-**e.** The diversity of T cell repertoires in all three groups was analyzed as described in the Methods. The diversity indexes of the T cell clones, including Shannon entropy **c.**, Simpson dominance **d.** and the Berger-Parker index **e.**, were all significantly decreased in the AS plaques, suggesting that the diversity of T cell repertoire in AS plaques was less than that in the other two groups. *: *p* < 0.05 *vs*. other two groups.

The proportion of in-frame (express functional TCR proteins) and out-of-frame (not express functional TCR proteins) clones directly impacts the function and composition of T-cell repertoire. The proportion of in-frame (out-of-frame) T cell clones in AS plaques was significantly higher (lower) than that in the peripheral blood of healthy subjects and patients with AS (*p* < 0.05) (Figure 3a and 3b). Additionally, there was no significant difference in the nucleotide insertion in TCR CDR3 regions of the T cell clones among all groups, which suggested that the diversity differences of the T-cell repertoires between the groups might not be due to the nucleotide insertion in CDR3. The insertion lengths in the TCR CDR3 were mainly < 22 nucleotides in all groups (Supplementary Figure S2a). The utilization of different amino acids in the TCR proteins is also one of the characteristics of T cell clonal diversity and closely associated with many diseases. However, there were no significant differences in the utilization rates of 20 common amino acids in the TCR proteins among the individuals of all three groups (Supplementary Figure S2b).

### Significant changes in diversity and V-J gene utilization of T cell clones in AS plaques

**Figure 4:**
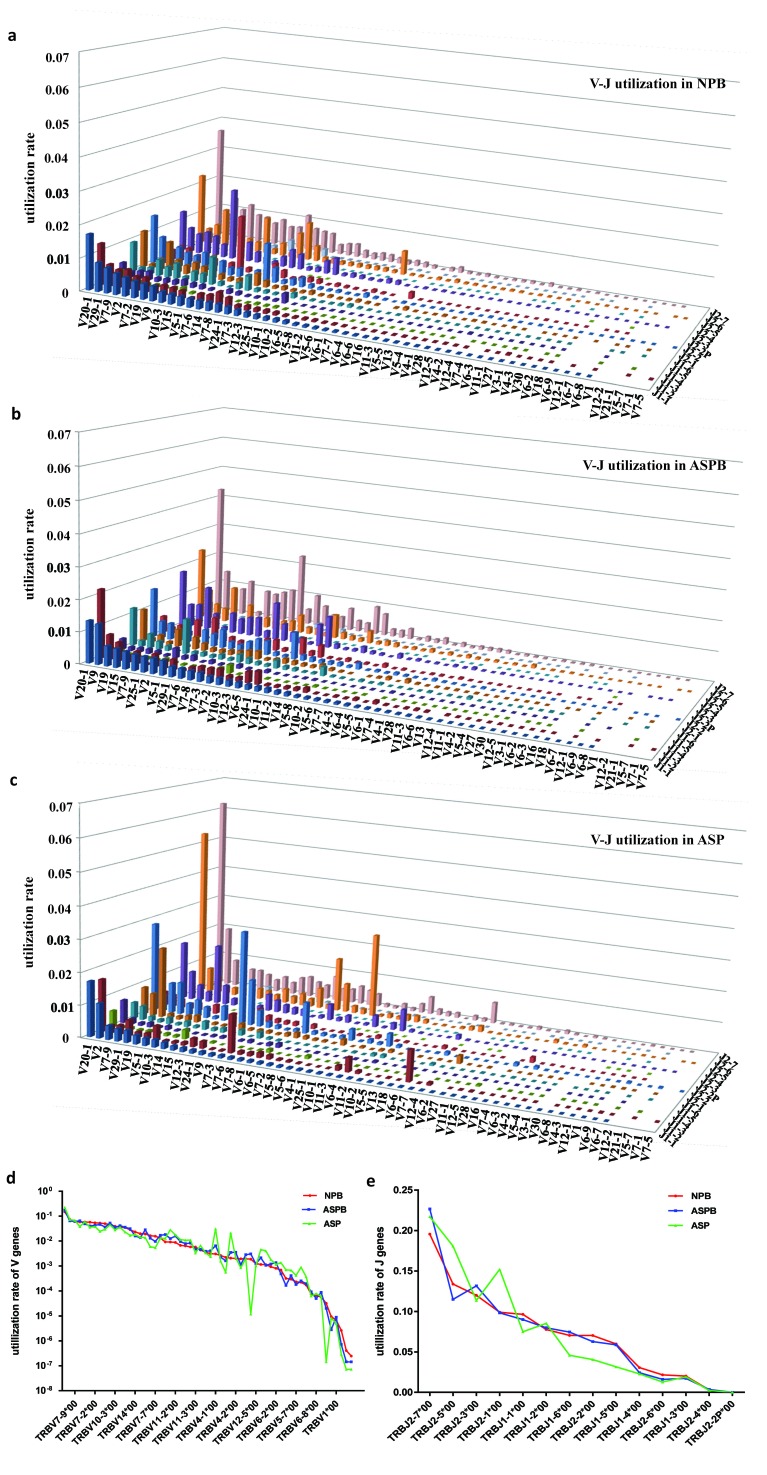
Significant changes in V-J gene utilization of T cell repertoires in AS plaques **a.**-**c.** The utilization rates of the V-J genes in all three groups. There were no notable differences in the overall profiles of the V-J gene utilization of T cell repertoires in the peripheral blood between normal subjects **a.** and patients with AS **b.**. The V-J gene utilization of the T cells clones in the AS plaques **c.** was obviously reduced, and high-frequency clones were markedly increased. **d.** and **e.** The distribution profiles of the utilization of V genes **d.** or J genes **e.** were essentially similar in all three groups. However, the utilization of some V genes (such as TRBV4-1, TRBV5-4, TRBV12-4) and J genes (such as TRBJ2-5, TRBJ2-1, TRBJ1-6) in the AS plaques was notably different from those in the other two groups.

The clonal diversity is one of the most important features of the T cell immune repertoire and reflects the function of the immune system. The diversity indexes of the T cell clones were significantly decreased in the AS plaques (*p* < 0.05) (Figure 3c-3e). Moreover, the overall profiles of the V-J gene utilization of T cell repertoires were similar in the peripheral blood between patients with AS and healthy subjects, but it was obviously reduced in the plaques. Namely, the types of V-J genes recombined by V and J genes in the AS plaques were fewer than that in the other two groups, which was consistent with the reduced diversity of the T cell clones in the AS plaques. However, the high-frequency T cell clones in AS plaques was markedly elevated compared to that in the other two groups, such as V20-1-J2-7 and V20-1-J2-5 (Figure 4a-4c). These results indicated that there might be some antigenic or pathological stimuli, which could activate and expand the corresponding T-cell clones to became relatively prominent. Additionally, the distribution trends of the utilization of the V or J genes of the T cell clones were similar in all the three groups. However, the utilization rates of some V genes (such as TRBV4-1, TRBV5-4, TRBV12-4) and J genes (such as TRBJ2-5, TRBJ2-1, TRBJ1-6) in the AS plaques were notably different from those in the other two groups (Figure 4d and 4e).

### PCR verification of the significantly expanded T cell clones in the AS plaques

**Figure 5:**
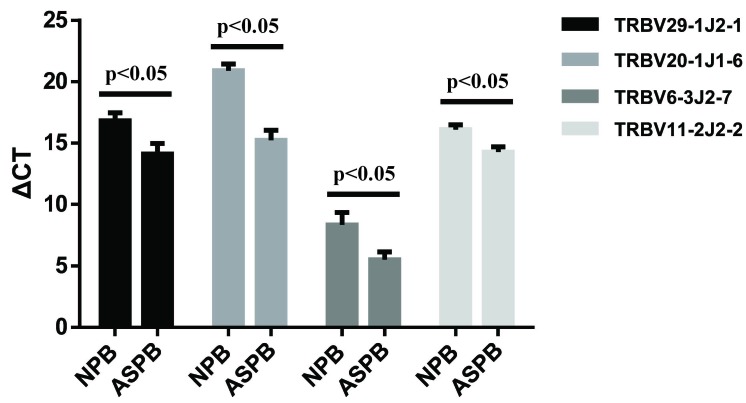
PCR verification of the significantly expanded T cell clones in the AS plaques The CDR3 sequences of six abnormally expanded T-cell clones with high-frequency in the peripheral blood and plaques of patients with AS were selected, and their ΔCT values were calculated after qPCR in the peripheral blood of patients with AS (ASPB) and normal subjects (NPB). The expression of four T cell clones (V29-1J2-1, V20-1J1-6, V6-3J2-7 and V11-2J2-2) in the ASPB group was significantly higher than that in the normal subjects (NPB) (*p* < 0.05), *n* = 11. All experiments were independently repeated 3 times.

To validate the T-cell clonotypes that were significantly expanded in the atherosclerotic plaques and peripheral blood of patients with AS compared to healthy subjects, six clones (V29-1J2-1, V20-1J1-6, V6-3J2-7, V7-6J2-3, V14J2-7 and V11-2J2-2) with high frequencies from 0.06% to 0.35% were selected and validated using real-time PCR. The results showed that the quantities or frequencies of the V29-1J2-1, V20-1J1-6, V6-3J2-7 and V11-2J2-2 clones in the peripheral blood of patients with AS were significantly higher than that of normal subjects (*p* < 0.05) (Figure 5).

## DISCUSSION

T cells are key regulators of immune responses and play important roles in the development of many diseases. The functions of T cells are closely related to the TCRs [14, 15]. To recognize the almost infinite number of xenogeneic/pathogenic antigens, the composition of the TCR repertoire must be sufficiently diverse [16]. This diversity is generated by the rearrangement of the V, D, and J genes in the TCR α, β, γ and δ loci, then the original T cell clones are further shaped by antigen-driven immune and inflammatory responses to form actual TCR repertoires [17]. Therefore, the composition and structure of the TCR repertoire not only directly affects the function of T cells but also reflects the interaction between genetic and environmental factors. The abnormalities of TCR repertoires are often associated with the development of many immune diseases [18]. In the peripheral T cells of patients with T2DM, the utilization of the V or J genes (especially TRBV7-8) and the amino acid lengths of the TCR CDR3 regions were significantly altered. These alterations were closely associated with an increased risk of diabetes [19]. Our study also found that the utilization of the V and/or J genes was altered in patients with AS, suggesting the relationship between TCR repertoires and atherosclerosis development.

Dysfunctions of immunity regulated by T lymphocytes are involved throughout the development of atherosclerosis [20]. Many local autoantigens contribute to these immunoregulatory abnormalities, resulting in the clonal restriction of T cells [2]. This restriction is associated with the plaque instability and inflammatory response [21-23]. In atherosclerotic lesions, ox-LDL specific T cells and antibodies and the accumulation of oligoclonal T cells were identified [24, 25]. For instance, T cells containing the TCR-V6 gene were expanded in atherosclerotic plaques and closely related to the recognition of ox-LDL, which suggested that ox-LDL was one of the autoantigens that induced strong, local T cell responses in plaques [26]. These T cell clones activated by corresponding specific antigens including ox-LDL can expand and produce various cytokines and bioactive molecules, thus modulating inflammatory responses and participating in the development of AS [24]. In present study, the T-cell clonotypes containing TCR-V6 gene were also identified, indicating that the T cell clones expanded in plaques may have the similar functions.

To date, the composition and structural features of the TCR repertoire in patients with AS are still not completely understood. Using the spectratyping technique, previous studies have determined that the CDR3 lengths of the TRBV genes are unrestricted in atherogenesis [27]. Several specific T cell clonotypes are expanded in unstable plaques of patients with AS, which suggests a specific, antigen-driven recruitment of oligoclonal T cells in unstable lesions [22]. In previous studies, 12 clonotypes (V18J2.3, V05J2.7, V25J2.6, V27J2.7, V24J2.3, V24J2.5, V15J2.4, V19J2.5, V04J2.3, V05J2.5, V24J2.4 and V20J2.6) expanded in patients with AS have been reported [28]. Because of the ultra-high sensitivity of IR-Seq, all the T cell clonotypes (including the abnormal subsets in patients with AS) detected in previous studies can be found in present study. Additionally, hundreds of abnormally expanded clonotypes (such as V29-1J2-1, V20-1J1-6, V6-3J2-7 and V11-2J2-2) were first found in our study. Recently, a study using Multi-N-plex PCR revealed a significant reduction of the TCR diversity in thrombosis and the peripheral blood of patients with AS compared with healthy controls [28]. However, our results showed that although the TCR diversity in the peripheral blood of patients with AS was decreased compared with that of healthy subjects, it was not significant. These contradictory results may be due to the fact that the resolving power of the methods used in the two studies is different. In this previous study, the analysis method of T cell receptor diversity based on the sizes of multiplex PCR products did not meet the requirements for revealing the millions of actual V(D)J genes. In present study, more than one million combinations of the V(D)J genes (namely clones) were detected by IR-Seq, which possibly reflected the more actual TCR repertoires. In addition, the difference in population between the two studies (Chinese Han population *vs*. European Caucasian population) might also explain the inconsistent results.

The numbers and types of T cell clones containing different TCR amino acid/nucleic acid sequences are extremely large (more than several millions). Many of the techniques used in most of the previous studies are difficult to accurately reveal the features of the numerous T cell subsets/clones and provide fine-tuned descriptions of the actual TCR repertoires [12, 29]. Many molecular characteristics of TCR repertoires, such as clonotype frequency, CDR3 diversity and CDR3 sequence analysis are needed to understand the mechanism of abnormal inflammation of atherosclerosis. The IR-Seq technology used in our study is based on the multiplex PCR and NGS and, to a large extent, can solve the above problems [30, 31]. Our study found that many molecular characteristics of the TCR β repertoire in AS plaques were significantly changed, particularly the reduced utility of VJ genes and the expansion of some clonotypes including V29-1J2-1, V11-2J2-2, V20-1J1-6 and V6-3J2-7. These results indicated that some antigens might shape the T cell repertoires and result in the expansion and activation of the T cell clones that recognize these antigens, which could lead to a series of immune responses. The isolation of these T cell clones can help us identify the corresponding antigens and understand the initiating mechanism of inflammatory response in AS development.

To our knowledge, the present study was the first to analyze TCR β repertoire in AS using IR-Seq with single-base resolution. Our results showed that compared to those in the peripheral blood of healthy subjects and patients with AS, the type of T cell clones were significantly reduced while the percentage of common clones (especially the high-frequency common clones) were elevated in the atherosclerotic plaques. The V and/or J gene utilization of T cell clones in the AS plaques was markedly decreased, resulting in the reduced diversity of T cell clonotypes. Meanwhile, several T cell clonotypes were significantly expanded in patients with AS. These results could enrich our understanding of the mechanism of AS development from the viewpoint of innate and adaptive immunity.

## MATERIALS AND METHODS

### Ethics approval and consent to participate

The present study has been approved by the Ethics Committee of Qilu Hospital and conformed to the Declaration of Helsinki. Informed consent was obtained from all subjects.

### Samples, genomic DNA isolation and multiplex polymerase chain reactions

All atherosclerotic plaques were collected from autopsy specimens. Samples of peripheral blood (5 mL each) from 55 patients who met the diagnostic criteria of atherosclerosis were obtained by venipuncture. Peripheral blood samples from 56 healthy subjects were obtained as controls. Peripheral blood mononuclear cells (PBMCs) were isolated from the peripheral blood by Ficoll density gradient centrifugation. The genomic DNA of all samples was purified using a genomic DNA extraction kit (Qiagen). The same amount of gDNA from each sample in the corresponding group was mixed and comprised the sample pools of atherosclerotic patients’ blood, healthy blood and atherosclerotic plaques. The TCRβ CDR3 regions were amplified from genomic DNA by multiplex polymerase chain reaction (PCR) with the specifically designed primer sets for the TCRβ CDR3 V and J regions (BGI).

### High-throughput sequencing

The products (100-190bp) of the multiplex PCR were purified using the QIAquick PCR Purification Kit (Qiagen). After end-repair, dA-tailing, adapter ligation and PCR amplification, the specific DNA fragments (200-314bp) were selected and purified. Then, the DNA fragments were subjected to library construction. Subsequently, the library quality (insert fragment length and library concentration) was evaluated by the Agilent 2100 Bioanalyzer and ABI StepOnePlus Real-Time PCR System. After bridge PCR amplification, the paired-end (PE) 101bp sequencing was performed on Hiseq2000 sequencing platform (Illumina). The detailed protocols were described in Additional file 1.

### Bioinformatics analysis

The raw sequencing data were filtered according to the following criteria: (1) reads contaminated by adapter sequences; (2) reads with more than 5% uncalled bases (N); (3) reads with an average quality score lower than 15 (based on the Illumina 0-41 quality system); and (4) PE reads with low-quality base readings (Q-score < 10) at the ends of reads or short reads (length < 60 bp). After filtration, sequence alignment was performed using miXCR [32]. The detailed protocols were described in Additional file 1. Other analyses, such as frequency interval distribution, cumulative frequency distribution and VDJ gene utilization, were conducted using homemade scripts. The diversity indexes (Shannon’s index, Simpson’s index and Berger- Parker index) were calculated using the diversity calculator from BPMSG website [33].

### Real-time PCR

The purified gDNA from each group was quantified by a Nanodrop 2000. Then, real-time PCR was performed using SYBR Green Master Mix (Takara) on a Bio-Rad CFX96. β-actin was used as an internal control. The primers were listed in Additional File Supplementary Table S3.

### Statistical analysis

The data were presented as the mean+SEM and compared using two-tailed Student’s *t*-tests or one-way analysis of variance with appropriate post-tests. *P* < 0.05 was considered statistically significant. The statistical analyses were conducted with GraphPad Prism software (GraphPad Software).

## SUPPLEMENTARY MATERIALS FIGURE AND TABLES









## Abbreviations

ACPA: Anti-citrullinated protein/peptide antibodies
AS: Atherosclerosis
CDR3: Complement determining region 3
IMGT: The international ImMunoGeneTics information system
IR-Seq: Immune repertoire sequencing
NGS: Next-generation sequencing
PBMCs: Peripheral blood mononuclear cells
PE: Paired-end
qRT-PCR: Quantitative real time polymerase chain reaction
RACE: Rapid amplification of cDNA ends
TCR: T cell receptor
T2DM: Diabetes mellitus type 2
TRBV: T cell receptor beta variable
