# Supplementary material for: Deep sequencing of the T cell receptor β repertoire reveals signature patterns and clonal drift in atherosclerotic plaques and patients
Source: Oncotarget. 2017 Aug 3;8(59):99312–22. doi: 10.18632/oncotarget.19892 (PMC5725094; doi:10.18632/oncotarget.19892)
Supplement: Supplementary file 1 [file oncotarget-08-99312-s001.pdf]

Deep sequencing of the T cell receptor β repertoire reveals signature patterns and clonal drift in atherosclerotic plaques and patients

SUPPLEMENTARY MATERIALS

Sample processing

1. Library construction

The qualified genomic DNA (gDNA) was prepared to construct the sequencing library for Illumina HiSeq 2000/2500. The TCRβ CDR3 regions were amplified from gDNA by multiplex PCR with the specifically designed primer sets for the V and J gene regions of TCRβ CDR3 (BGI) using QIAGEN multiplex PCR Kit (30 cycles). The amplicons were separated by electrophoresis on a polyacrylamide gel, and the fraction of 100-190 bp was excised and purified using QIAquick PCR Purification Kit. After end-repair, dA-tailing, adapter ligation and PCR amplification, the specific DNA fragments (200-314 bp) were selected and purified. Then, the DNA fragments were tagged with the library barcodes.

2. Validation of the library quality

The library quality (insert fragment length and library concentration) was evaluated by two methods. The Agilent 2100 Bioanalyzer (Agilent DNA 1000 Reagents) was used to detect the insertion range of the library. The ABI StepOnePlus Real-Time PCR System (TaqMan Probe) was used to detect the library concentration.

3. Paired-end sequencing

The qualified libraries were denatured into a single chain by adding NaOH and diluted to a certain concentration according to the expected amount of data. Then, the libraries were added to the FlowCell and hybridized with the linker on the FlowCell. Subsequently, the bridge PCR was performed on the cluster generation platform cBot. Finally, the prepared FlowCell was sequenced by HiSeq 2000 sequencing system. The sequencing type was PE101.

Data analysis

1. Filtration of raw sequencing data

The sequencing reads were trimmed and the reads that meets the following criteria are filtered. (1) reads contaminated by adapter sequences; (2) reads with more than 5% uncalled bases (N); (3) reads with an average quality score lower than 15 (based on the Illumina 0-41 quality system); (4) PE reads with low-quality base readings (Q-score <10) at the ends of reads or short reads (length <60 bp).

2. Extraction of the sequence of CDR3 region

The clean sequencing data after filtration were aligned via miXCR to the reference sequences of V, D, J and C genes of T-cell receptors. Then the aligned sequences were assembled in order to extract specific gene regions e.g. CDR3. The results of alignment and assembly were exported to readable text files which contain the information about all clonotypes (clone abundance, CDR3 sequence, V, D, J genes, etc.). Subsequently, many important features of the TCRβ clonotypes were analyzed using homemade script and Microsoft Excel software. The features included clone types, clone frequency, fixed frequency, nucleotide insert, cumulative frequency, length distribution of total and common T-cell clones at nucleotide and amino acid levels, open reading frame and amino acid sequences. The diversity of TCRβ clonotypes was indicated by three diversity indexes, namely Shannon's index, Simpson's index and the Berger-Parker index. They were calculated using the diversity calculator from the BPMSG website. The subgroup classification and utilization of V and J genes were obtained by miTCR software and the statistical analysis of VJ genes combinations, V or J gene utilization was conducted with GraphPad Prism software and Excel. The changed VJ clones in different groups were screened by Excel and ACCESS software.

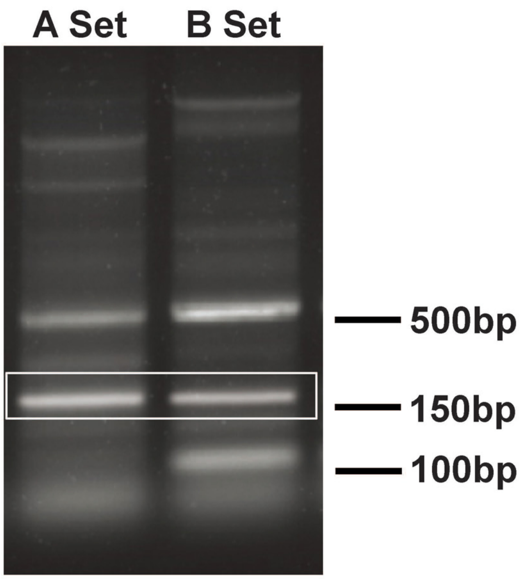

Supplementary Figure 1: The representative electrophoresis result of the multiple-PCR products of T cell CDR3 region

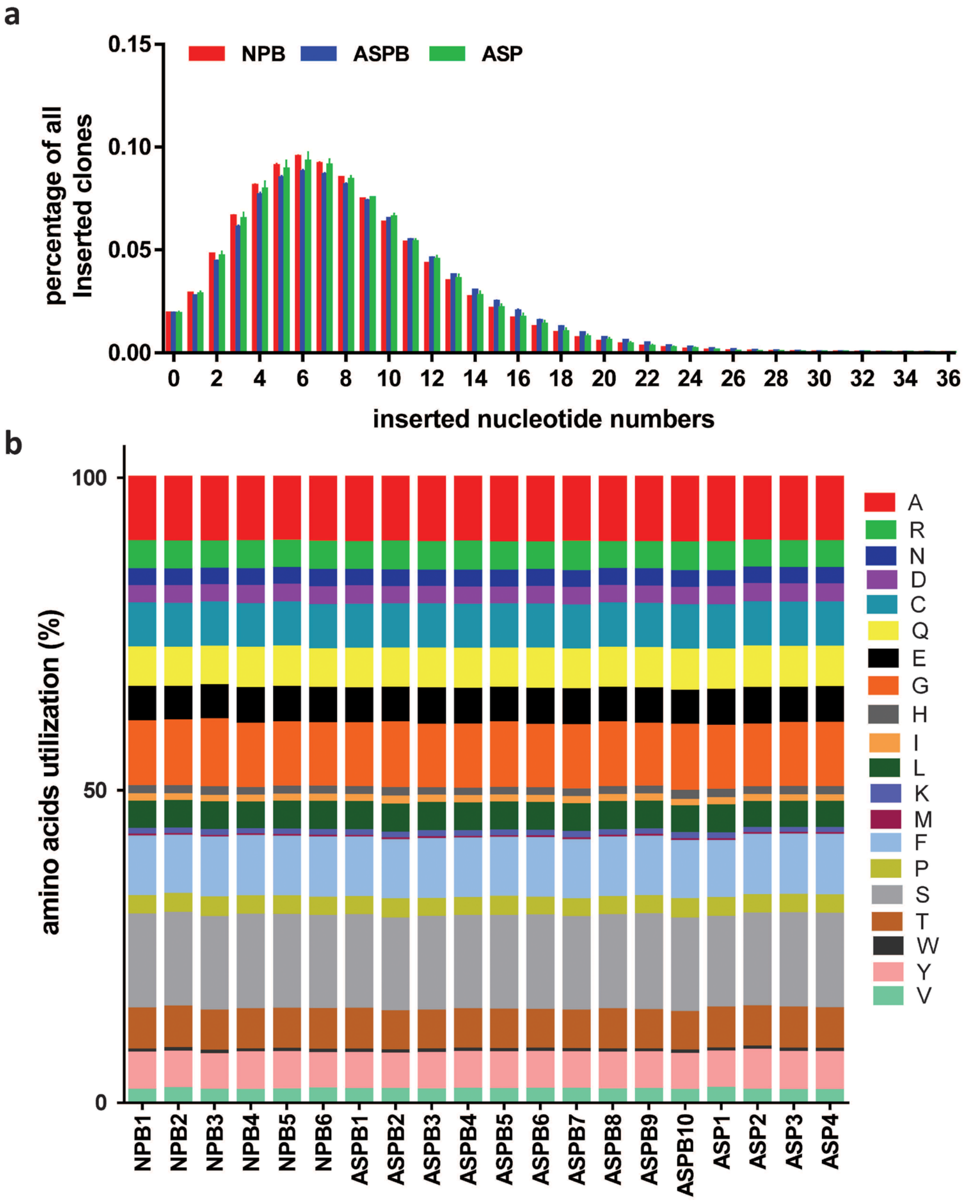

Supplementary Figure 1: The representative electrophoresis result of the multiple-PCR products of T cell CDR3 region

For Supplementary Tables see in Supplementary Files
