# Supplementary material for: Deep sequencing of the T cell receptor β repertoire reveals signature patterns and clonal drift in atherosclerotic plaques and patients
Source: Oncotarget. 2017 Aug 3;8(59):99312–22. doi: 10.18632/oncotarget.19892 (PMC5725094; doi:10.18632/oncotarget.19892)
Supplement: Supplementary file 2 [file oncotarget-08-99312-s002.docx]

**Additional Files**

**Supplementary Table 1: Clinical characteristics of the study groups**

|  | NPB pool (n=56) | ASPB pool (n=55) | ASP pool (n=4) |
| --- | --- | --- | --- |
| Age (mean ± SD)  Male (%)  Major risk factors, n (%)  Hyperlipidemia  Hypertension  Diabetes  Smoking  Drinking | 60±7  35 (63%)  32 (57%)  14 (25%)  18 (32%)  18 (32%)  17 (30%) | 61±8  39 (71%)  23 (42%)  16 (29%)  32 (58%)  24 (43%)  15 (27%) | 57±2  3 (75%)  2 (50%)  1 (25%)  1 (25%)  2 (50%)  2 (50%) |

**NPB:** normal peripheral blood; **ASPB:** atherosclerotic peripheral blood; **ASP:** atherosclerotic plaque
