# Supplementary material for: Deep sequencing of the T cell receptor β repertoire reveals signature patterns and clonal drift in atherosclerotic plaques and patients
Source: Oncotarget. 2017 Aug 3;8(59):99312–22. doi: 10.18632/oncotarget.19892 (PMC5725094; doi:10.18632/oncotarget.19892)
Supplement: Supplementary file 3 [file oncotarget-08-99312-s003.docx]

**Supplementary Table 2: The quality information about the Seq-data of each sample**

| Samples | Clean reads | Unique reads  /clonotypes | Max.  reads | Mean reads per clonotype | Alignment rate with IMGT | Q30 |
| --- | --- | --- | --- | --- | --- | --- |
| ASPB pool | 14059561 | 89229 | 227700 | 157 | 91% | >90% |
| ASPB individual |  |  |  |  |  |  |
| 1 | 20981392 | 65956 | 995500 | 318 | 91% | >90% |
| 2 | 17473922 | 75127 | 1218000 | 232 | 93% | >90% |
| 3 | 17924217 | 54002 | 1915000 | 331 | 92% | >90% |
| 4 | 13318421 | 75168 | 894400 | 177 | 80% | >90% |
| 5 | 14715559 | 61584 | 1632000 | 239 | 91% | >90% |
| 6 | 14827490 | 66213 | 233300 | 223 | 93% | >90% |
| 7 | 11752652 | 63121 | 1746000 | 186 | 95% | >90% |
| 8 | 11349123 | 58674 | 896200 | 193 | 94% | >90% |
| 9 | 17038250 | 75683 | 240100 | 225 | 92% | >90% |
| NBP pool | 12190154 | 104850 | 214700 | 116 | 87% | >90% |
| NBP individual |  |  |  |  |  |  |
| 1 | 14011117 | 140323 | 2036000 | 99 | 89% | >90% |
| 2 | 13546266 | 152946 | 1407000 | 88 | 89% | >90% |
| 3 | 15424253 | 191774 | 469200 | 80 | 92% | >90% |
| 4 | 15301412 | 132940 | 1265000 | 115 | 92% | >90% |
| 5 | 14465956 | 150458 | 901400 | 96 | 91% | >90% |
| ASP pool | 13549316 | 31405 | 365600 | 431 | 92% | >90% |
| ASP individual |  |  |  |  |  |  |
| 1 | 9956776 | 77887 | 168600 | 127 | 85% | >90% |
| 2 | 11121549 | 88100 | 791800 | 126 | 82% | >90% |
| 3 | 10087751 | 98330 | 729700 | 102 | 80% | >90% |
