# Supplementary material for: Deep sequencing of the T cell receptor β repertoire reveals signature patterns and clonal drift in atherosclerotic plaques and patients
Source: Oncotarget. 2017 Aug 3;8(59):99312–22. doi: 10.18632/oncotarget.19892 (PMC5725094; doi:10.18632/oncotarget.19892)
Supplement: Supplementary file 4 [file oncotarget-08-99312-s004.docx]

**Supplementary Table 3: The primers of TCR clonotypes to be verified and β-actin**

| Gene | Forward (5' -> 3') | Reverse (5' -> 3') |
| --- | --- | --- |
| V29-1J2-1 | TCATTGACAAGTTTCCCATC | GAAGAACTGCTCATTGTAGG |
| V20-1J1-6 | AAGCCTGACCTTGTCCACTC | TACTGCGTATCTGTTCGAGG |
| V7-6J2-3 | GCAGATGACTCAGGGCTGCC | TACTGCGTATCCCCTGGGCT |
| V14J2-7 | TTCTTAGCTGAAAGGACTGG | ACTGTCTAGCCCTGTCTTGG |
| V11-2J2-2 | GGATGATTCACAGTTGCCTA | CAACTAAGCTGCTGGCACAG |
| V6-3J2-7 | AGAGGTCCCTGATGGCTACAAT | ACTGCTCGTAGGAGTAACTGCT |
| β-actin | ACGGGGTCACCCACACTGTGCCCATCTA | AGAAGCATTTGCGGTGGACGATGGAGGG |
